# Respiratory Manifestations of the Activated Phosphoinositide 3-Kinase Delta Syndrome

**DOI:** 10.3389/fimmu.2018.00338

**Published:** 2018-03-05

**Authors:** Alison M. Condliffe, Anita Chandra

**Affiliations:** ^1^Department of Infection, Immunity & Cardiovascular Disease, University of Sheffield, Sheffield, United Kingdom; ^2^Department of Medicine, University of Cambridge, Cambridge, United Kingdom; ^3^Laboratory of Lymphocyte Signalling and Development, Babraham Institute, Cambridge, United Kingdom

**Keywords:** activated phosphoinositide 3-kinase delta syndrome, respiratory infection, pneumonia, bronchiectasis, antibody deficiency, lymphoproliferation

## Abstract

The activated phosphoinositide 3-kinase δ syndrome (APDS), also known as p110δ-activating mutation causing senescent T cells, lymphadenopathy, and immunodeficiency (PASLI), is a combined immunodeficiency syndrome caused by gain-of-function mutations in the phosphoinositide 3-kinase (PI3K) genes *PIK3CD* (encoding p110δ: APDS1 or PASLI-CD) and *PIK3R1* (encoding p85α: APDS2 or PASLI-R1). While the disease is clinically heterogeneous, respiratory symptoms and complications are near universal and often severe. Infections of the ears, sinuses, and upper and lower respiratory tracts are the earliest and most frequent manifestation of APDS, secondary to both respiratory viruses and to bacterial pathogens typical of defective B cell function. End organ damage in the form of small airways disease and bronchiectasis frequently complicates APDS, but despite documented T cell defects, opportunistic infections have rarely been observed. Antimicrobial (principally antibiotic) prophylaxis and/or immunoglobulin replacement have been widely used to reduce the frequency and severity of respiratory infection in APDS, but outcome data to confirm the efficacy of these interventions are limited. Despite these measures, APDS patients are often afflicted by benign lymphoproliferative disease, which may present in the respiratory system as tonsillar/adenoidal enlargement, mediastinal lymphadenopathy, or mucosal nodular lymphoid hyperplasia, potentially causing airways obstruction and compounding the infection phenotype. Treatment with rapamycin and PI3Kδ inhibitors has been reported to be of benefit in benign lymphoproliferation, but hematopoietic stem cell transplantation (ideally undertaken before permanent airway damage is established) remains the only curative treatment for APDS.

## Introduction

Following the initial description in 2013 of gain-of-function (GOF) mutations resulting in enhanced phosphoinositide 3-kinase (PI3K) δ signaling as the cause of a combined immune deficiency syndrome [the activated phosphoinositide 3-kinase δ *s*yndrome (APDS), also known as p110δ-*a*ctivating mutation causing *s*enescent T cells, *l*ymphadenopathy, and *i*mmunodeficiency (PASLI)], multiple case reports and several case series have highlighted the protean clinical feature of this newly recognized disease. The first reports (1–3) identified mutations in the gene (*PIK3CD*) encoding the p110δ catalytic subunit of PI3Kδ, and several additional GOF mutations have since been described [e.g., Ref. (4–9)]. Subsequently, patients with a highly reminiscent clinical phenotype who did not harbor APDS-associated *PIK3CD* mutations were found instead to have exon-skipping mutations in the Class 1A regulatory PI3K subunit p85α encoded by *PIK3R1* [e.g., Ref. (10–16)]; these mutations disrupt the inhibitory interactions with the catalytic subunit of PI3Kδ (17), increasing both basal and stimulated activation. The resulting clinical syndrome, termed APDS2 (or PASLI-R1), phenocopies many of the APDS1 disease manifestations but with a higher incidence of growth retardation and in some cases, overlap with SHORT syndrome [short stature, hyperextensibility, hernia, ocular depression, Rieger anomaly, and teething delay (14, 18)]. More recently, four patients with mutations leading to haploinsufficiency of PTEN (a lipid phosphatase that opposes PI3K activation) have been found to have immunodeficiency with an APDS-like syndrome (9, 19). Despite the different genetic underpinnings, the clinical features have marked similarities; a recurring theme is that respiratory manifestation (predominantly infections but also non-infectious complications) affect the majority of patients, occur early in the course of the disease, and are challenging to manage clinically.

## Respiratory Infections in APDS

### Incidence and Age of Onset

While a few isolated cases have been identified who are completely asymptomatic (20) or who have severe extrapulmonary manifestations but minimal or no respiratory symptomatology (21), recurrent respiratory tract infections are reported near universally in APDS; indeed, they may be the sole manifestation of the disease (16), and they may be both very frequent and severe (5). Unfortunately, however, differences in definitions and nomenclature make direct comparisons between published studies challenging at times. For example, Coulter et al. (20) reported that 51 (98%) of a cohort of 53 patients with APDS1 suffered recurrent respiratory infections, subdividing these episodes further into radiologically confirmed pneumonia (85%), recurrent otitis media (49%, severe enough to cause permanent hearing loss in 8% of the total), chronic rhinosinusitis (45%), and tonsillitis (28%). By contrast, in their description of 36 patients with APDS2, Elkaim et al. (22) noted recurrent upper respiratory tract infections (including both otitis media and sinusitis in this definition) in 100% of cases, and lower respiratory infections (defined as either bronchitis or pneumonitis) in 70% of their cohort, without further breakdown. A recently published Dutch cohort (8) reporting 13 newly identified patients (11 with APDS1 and 2 with APDS2) stated that all had both upper and lower respiratory tract infections but did not supply further clinical details as the focus of the manuscript was B cell differentiation and maturation. A Chinese case series of 15 APDS1 patients (23) reported pneumonia had been diagnosed in 12 of the cases (80%).

In addition to the high frequency of such infections, their onset is early in life [10 months–10 years (22) and <1–7 years (20)] and is the commonest reason for presentation to medical/immunological services. Even in patients whose presentation is precipitated by other acute manifestations [e.g., intussusception (24) or gut-associated T cell lymphoproliferation (25)], a retrospective history of recurrent respiratory infections is usually present. Thus, although precise definitions vary between studies, it is possible to conclude that APDS patients suffer early, frequent, and severe respiratory infections. This concurs with the accompanying article presenting initial data collected by the ESID APDS registry (Maccari et al., personal communication^1^).

Despite these broad similarities, the severity and pattern of infections (as well as other manifestations) varies considerably between individual patients, even when grouped according to genotype and even within affected family members. In one E1021K APDS1 kindred (26) in which three individual affected family members exhibited a mild, intermediate, and severe spectrum respiratory infections, there seemed to be a broad association of severer phenotype with more suppressed IgG and lower class-switched memory B cells. However, this correlation was not observed in other affected families (2, 27) and does not seem to be recapitulated in larger cohort studies. To date, no circulating biomarker has been reliably linked to respiratory phenotypes, but larger longitudinal studies may enable such correlation to be identified in future.

### Microbiology of Infections

While some microbiological data have been published, milder infections are generally self-reported and not supported by identification of a causal pathogen. It is therefore likely that most of the reported isolates are derived from infections at the severer end of the spectrum, in particular those requiring consultation with health-care professionals; this could skew the available data.

#### Bacterial Infections

**Figure 1:**
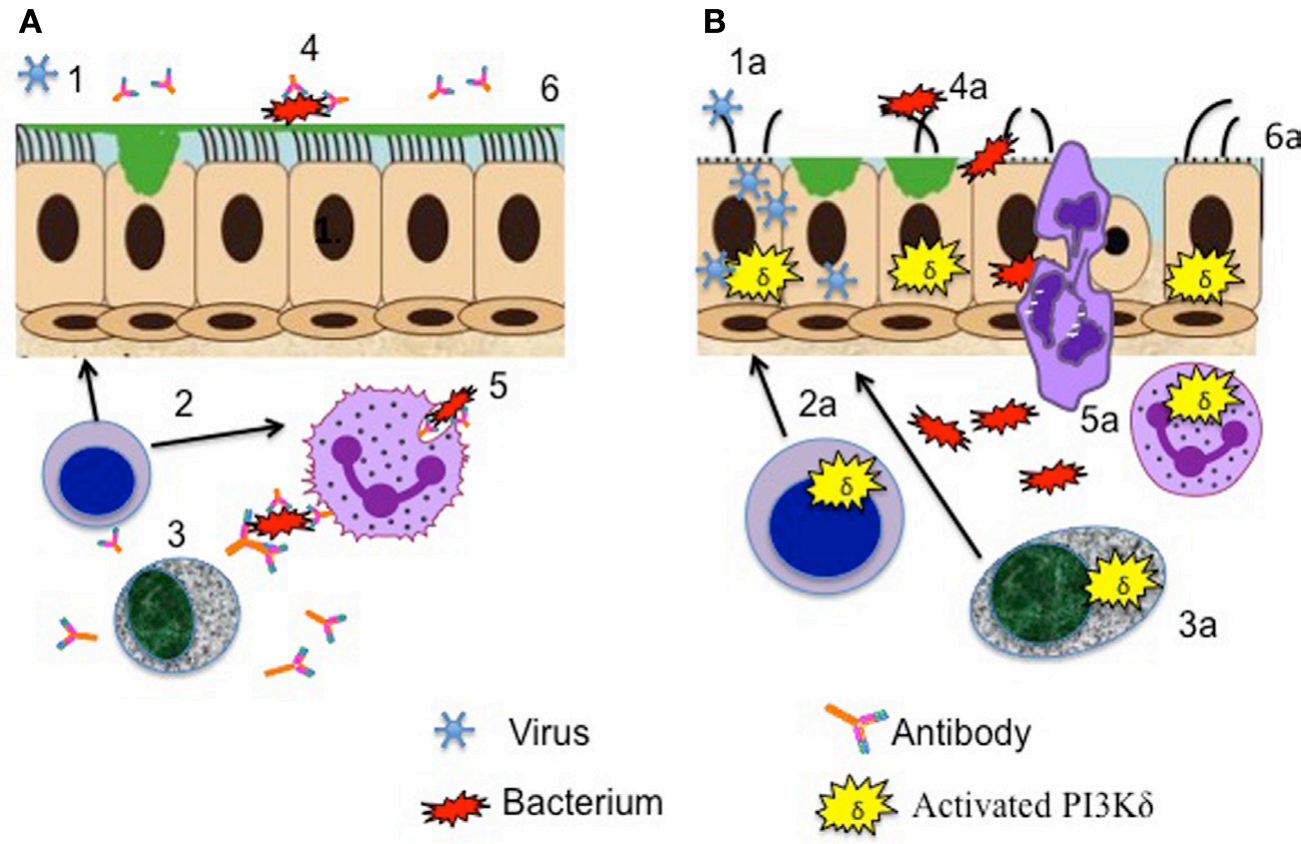
Aberrant cellular functions contributing to respiratory infection in activated phosphoinositide 3-kinase δ syndrome. **(A)** Healthy lung defenses. (1) Epithelial defenses counteract viral pathogens, aided by (2) effective T cells cytokine production. (3) Antibody production by B cells promotes (4) bacterial killing and (5) opsonophagocytosis. (6) Respiratory epithelial surfaces are preserved intact and continue to function to repulse invading pathogens. **(B)** Lung defenses compromised by activating mutations leading to enhanced phosphoinositide 3-kinase (PI3K) δ signaling. (1a) Viral entry and replication in airway epithelial cells are promoted, reducing barrier integrity. (2a) Aberrant cytokine production by T cells and (3a) failure of antibody production promote (4a) bacterial invasion with (5a) inadequate handling of pathogens by phagocytes. (6a) Repeated cycles of infection lead to long-term airway damage.

There is concordance that the commonest respiratory bacterial isolates are *Haemophilus influenzae* and *Streptococcus pneumoniae* (20, 22), *Staphylococcus aureus, Moraxella catarrhalis, Pseudomonas aeruginosa*, and *Klebsiella* species have also been reported (20, 28). This spectrum of pathogens is highly reminiscent of other primary antibody deficiency syndromes such as common variable immune deficiency. Defective antibody production (Figure 1) results in failure of antibody-mediated killing mechanisms such as opsonophagocytosis. However, abnormalities in immunoglobulin levels are heterogeneous in all of the published case series of APDS; Coulter et al. (20) reported that total IgG was reduced in just 43% of their APDS1 patient group, although defective class switch recombination and (when measured) specific antibody formation were more frequent; similarly, 50% of the Dutch APDS cohort had low IgG and high IgM levels (8). Hypogammaglobulinemia was more frequent (87%) in the APDS2 patients reported by Elkaim et al. (22). Interestingly, low IgG/IgA levels do not seem to reliably predict a more severe respiratory phenotype or correlate with the presence of bronchiectasis [for example, Coulter et al. (20) noted that 63% of patients with CT proven bronchiectasis had normal total IgG levels]. It is uncertain whether this lack of correlation of end organ damage with IgG reflects the widespread prevalence of more subtle antibody defects, additional aberrant B cell functions (e.g., abnormal cytokine production), the additive impact of the well-established abnormalities in T cell function (3) or other, as yet undetermined mechanisms. Of note, PI3Kδ inhibition reduced airway epithelial oxidative and endoplasmic reticulum stress in response to *Aspergillus fumigatus* exposure, both in cultured cells and in mouse lungs (29), suggesting that excessive PI3Kδ activity may be detrimental to local respiratory defenses as well as impairing adaptive immunity (Figure 1).

#### Viral Infections

The susceptibility of APDS patients to systemic infection with herpes viruses is well documented; however, they also seem to experience an excessive burden of respiratory viral infections. Coulter et al. (20) noted that significant adenovirus infections occurred in 17% of their APDS1 cohort, with adenovirus isolated from various sites including bronchoalveolar lavage fluid; other common viruses identified during respiratory exacerbations included respiratory syncytial virus (RSV), parainfluenza virus, and echovirus and coxsackie viruses (20). Significant RSV infections have also been noted by others [e.g., Ref. (14, 15)], and additionally a patient with pericarditis caused by echovirus infection has also been reported (30). While T cell-mediated antiviral mechanisms are undoubtedly compromised in APDS patients, it is worth reflecting that many viral pathogens subvert local host cell PI3K signaling (Figure 1). Herpesviruses in particular express multiple proteins that target PI3K/Akt to facilitate viral infection, replication, latency, and reactivation (31). Increased PI3Kα, rather than PI3Kδ expression and activity in primary bronchial epithelial cells isolated from patients with COPD, was found to underpin increased susceptibility to H3N2 and H1N1 influenza viral infection (32); inhibition of PI3K signaling restored protective antiviral responses and suppressed infection in this setting. It is plausible to extrapolate from these findings that excessive airway cell PI3K activity (whatever the isoform responsible) might predispose to airway viral invasion (Figure 1). With regard to APDS-relevant respiratory viral pathogens, the adenovirus E4-ORF1 (early region 4 open reading frame 1) protein enhances viral replication by activating PI3K (33). Likewise, infection with coxsackie virus activates PI3K/AKT signaling and suppression of these pathways diminished viral capsid protein expression and viral release (34), and PI3Kδ mediates dsRNA-induced upregulation of airway epithelial PD-L1, a co-inhibitory molecule associated with the escape of viruses from the mucosal immunity (35).

#### Mycobacterial and Fungal Infections

Although pulmonary mycobacterial infections have not been reported in APDS, local infection with Bacillus Calmette–Guérin (BCG) have been documented following vaccination (20), and in a separate study, a failure of patient-derived monocyte-derived macrophages to kill internalized BCG, restored by a PI3Kδ inbitor, was demonstrated (36). It would therefore seem prudent to ensure patients with APDS have sputum samples screened for mycobacteria as well as standard pathogens. To date, despite the marked T cell senescence that characterizes APDS, no patients with pulmonary pneumocystis pneumonia (PCP) or invasive aspergillosis have been reported, but interestingly one of two patients reported with a PTEN mutations causing an “APDS-like” syndrome contracted PCP at the age of 4 months and the other was reported to have suffered from “pulmonary aspergillosis,” although further details were not supplied (9). PI3Kδ activity supports neutrophil-mediated killing of *A. fumigatus* hyphae (37), and normal neutrophil PIP_3_ levels and oxidative burst were seen in response to soluble stimuli (2), hence increased susceptibility to this organism would not be predicted.

### Complications of Respiratory Infections in APDS

#### Bronchiectasis

**Figure 2:**
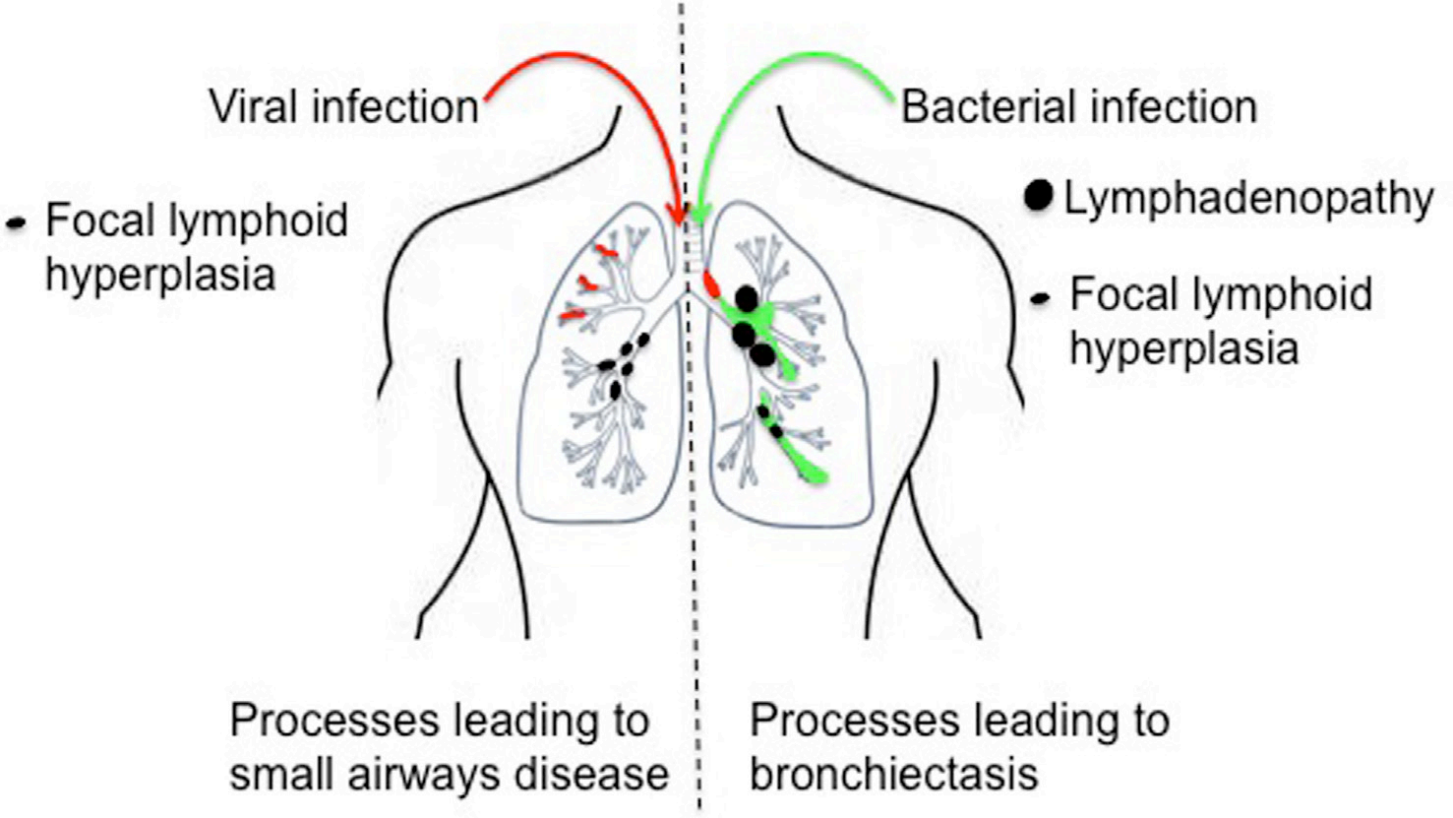
Processes leading to airway damage in activated phosphoinositide 3-kinase δ syndrome. Repeated episodes of viral bronchiolitis may lead to small airway damage and mosaic attenuation, compounded by local obstruction secondary to focal lymphoid hyperplasia. Recurrent bacterial infection leads to chronic inflammatory damage of the larger airways and the development of bronchiectasis; post-obstructive bronchiectasis may also occur secondary airway obstruction, which may be extra-luminal (intrathoracic lymphadenopathy) or intra-luminal (focal lymphoid hyperplasia).

Bronchiectasis (abnormal widening of the bronchi or their branches; Figure 2) is one of the commonest and most debilitating consequences of recurrent respiratory infection, and compounds the problem, increasing the host susceptibility to further lower respiratory tract infections and facilitating airway colonization with pathogenic bacteria (38, 39). A number of mechanisms may lead to the development of bronchiectasis in APDS. First, the frequent respiratory infections noted above may lead directly to airway damage, weakening the airway wall. Second, focal nodular lymphoid hyperplasia may be of sufficient magnitude to obstruct segmental or even lobar airways, potentially leading to post-obstructive bronchiectasis. Third, compromise of the adaptive immune response may predispose to bronchiectasis. Aberrant neutrophil function has been linked to bronchiectasis and correlates with disease severity and exacerbations (40, 41). Excessive (and perhaps dysregulated) neutrophil PI3K activation has been linked to airway damage in COPD (42), and inhibition of PI3K (using pan-PI3K inhibitors or inhibitors selective for PI3Kδ or PI3Kγ) was able to restore neutrophil migratory accuracy in both COPD and in the elderly (42, 43). Neutrophil function has been little studied in APDS: Angulo et al. (2) presented data from just *n* = 1–2 patients but did show an apparent reduction in neutrophil chemotaxis to IL-8 in cells derived from a patient with APDS; however, directionality and accuracy or migration were not assessed in this limited study. Further assessment of neutrophil function in APDS patients or in animal models of APDS would be of interest.

Recurrent respiratory infections precede a diagnosis of bronchi-ectasis by several years in most reported cases of APDS (see text footnote 1), but this apparent temporal progression may be confounded by delays in undertaking CT scans, and uptake of this investigation may vary between institutions and on a wider scale between countries. Earlier identification of patients and establishing treatment regimens including immunoglobulin replacement and antibiotic prophylaxis, or HSCT, might delay or prevent this complication, but to confirm this will require longitudinal observation. An early review (15) of 49 APDS1 and 15 APDS2 patients (all that had been published at the time of their review) suggested a higher incidence of bronchiectasis in APDS1 versus APDS2. In the most detailed study of bronchiectasis in APDS to date (20), CT chest scans from 31 patients with APDS1 were independently reviewed by 2 specialist thoracic radiologists; bronchiectasis was felt by both radiologists to be present in 21 of the 31 available scans (60%), with an average of three lobes affected. In one case, lobar consolidation was observed to progress to focal bronchiectasis, supporting a causal link between airway infection and airway wall damage. In contrast in a study of APDS2 (22), an incidence of just 18% bronchiectasis was found, but this study relied on the attending physician’s response to a questionnaire, and central review of scans was not undertaken. Could this reflect a true difference between APDS1 and APDS2? A lower incidence of bronchiectasis (only 2 of 10 APDS1 patients in whom CT scans were available were diagnosed with this condition) was noted in a smaller study (8) although bronchial wall thickening was highlighted in an additional four patients; neither of the APDS2 patients in this cohort had bronchiectasis. In a further case series (23), the reported incidence of bronchiectasis in APDS1 was just 5/15 (33%). Given the variability in chest CT uptake and reporting, it seems reasonable to conclude that bronchiectasis is a frequent complication of APDS, whatever the causal mutation; indeed initial data from the ESID APDS both APDS1 and APDS2 patients suggest an overall incidence of bronchiectasis of approximately 60%. Apparent differences between studies may reflect small sample sizes, geographical differences in CT uptake, and interindividual variation in CT reporting; however, larger cohort studies and longitudinal observation may be required to clarify this and exclude a genuine difference between APDS1 and APDS2.

#### Small Airways Disease

Bronchiectasis is an expected complication of recurrent bacterial respiratory infection and is well known to be associated with primary antibody deficiency. Unexpectedly, the commonest radiological abnormality (in 88%) flagged by specialist radiologists in the APDS1 cohort described by Coulter et al. (20) was not bronchiectasis or inflammatory change but mosaic attenuation, indicative of reduced perfusion of poorly ventilated lung regions. Air trapping (a related finding, secondary to airway obstruction) was also noted in 2/9 APDS1 patients in a separate study (8), and mosaic attenuation was flagged as a radiological feature of APDS1 but not enumerated by Angulo et al. (2). These more subtle CT abnormalities are likely to reflect the impact of recurrent episodes of viral bronchiolitis but could also be secondary to focal lymphoid hyperplasia (Figure 2 and see below). Further assessment of APDS patients for small airways disease using specialist pulmonary function methodologies (e.g., multi-breathe washout and forced oscillometry) or imaging modalities such as MRI with hyperpolarized helium or xenon might more accurately delineate this unexpectedly common radiological abnormality (44).

## Non-Infectious Respiratory Manifestations of APDS

### Benign Lymphoproliferation

Tonsillar and adenoidal hypertrophy is a frequent manifestation of APDS. A detailed analysis of this complication in APDS2 (22) revealed ear, nose, and throat chronic lymphoid hyperplasia without the need for surgical interventions in three (11%) patients, adenoidectomies, tonsillectomy, or both in seven (26%) patients and multiple surgical resections in three patients; one afflicted patient developed postoperative pharyngeal stenosis ultimately requiring tracheotomy. Coulter et al. (20) noted recurrent tonsillitis in 15/53 APDS1 patients (28%) with a need for tonsillectomy in 5/53 (13%) but listed this as an infectious rather than a lymphoproliferative complication. While occasional case reports have highlighted significant tonsillar hypertrophy in APDS1 (6, 24), it seems to be noted more frequently, and to be more severe in APDS2 [e.g., Ref. (12, 14, 27, 45)]. Tonsillar biopsies from two APDS2 patients demonstrated small B cell follicles rather than the atypical follicular hyperplasia reported in biopsies of lymph nodes/mucosal follicular hyperplasia from APDS1 (7, 20) and APDS2 (14), but other features such as reduced mantle layers and infiltration with PD1 +ve T cells were concordant, suggesting a related immunopathogenesis.

Benign lymphoproliferation has been widely reported in both APDS1 and 2, but in most cases mediastinal lymphadenopathy (which requires CT for ascertainment) is not separately reported. However, 16/31 APDS patients (20) were noted to have mediastinal lymphadenopathy, which was in a regional draining station to concurrent lobar consolidation in four instances, compatible with an infection-driven etiology. In the same study, 8 of 10 patients with persistent intrathoracic lymphadenopathy had bronchiectasis and recurrent consolidation, again suggesting a possible role for infection driving lymphoproliferation in this setting. In this study, 5/53 patients had mucosal nodular lymphoid hyperplasia identified bronchoscopically; the same phenomenon was observed in 6/9 of the APDS1 patients reported by Lucas et al. (3), all of whom underwent bronchoscopy, suggesting that milder cases will go undetected unless this invasive test is undertaken. As noted above, it is possible that this process contributes to the mosaic attenuation/air trapping noted on CT (Figure 2), and larger nodules might also lead to partial or total airway occlusion, segmental collapse, and post-obstructive bronchiectasis (Figure 2).

Of interest, although APDS can present with a CVID-like picture, it has not been associated with interstitial lymphoid or granulomatous infiltrates (granulomatous lymphocytic interstitial lung disease).

### Malignant Lymphoproliferation

Lymphoma has been reported to be a frequent complication of both APDS1 and APDS2 (20, 22, 30). The metabolic reprogramming that occurs during malignant transformation through the upregulation of aerobic glycolysis has been used to distinguish benign lymphoproliferation from malignant disease; this can be probed on positron emission tomography by the increased uptake of the glucose analog, ^18^F-fluorodeoxyglucose; biopsy is required where clinical or radiological suspicion is high. Lymphoma may involve mediastinal lymph nodes, or bronchus-associated lymphoid tissue, but this would normally be as part of a systemic process, and mediastinal nodes are more challenging to sample for histology than more peripheral nodes. While many lymph node stations in the chest are accessible *via* endobronchial ultrasound, and this technique has been used to diagnose lymphoma in immunocompetent patients (46), whole nodes cannot be removed in their entirety by this route; given the challenges in distinguishing between benign or malignant disease in immunodeficiency in general and APDS in particular, a larger pathological sample may be required. In this setting, if other nodes are not readily biopsied, a mediastinoscopy or video-assisted thoracoscopy might be required.

### Other Non-Infectious Complications

Although congenital abnormalities have been reported, most are extra-thoracic. One patient with APDS1 was diagnosed with a pulmonary sequestration requiring lobectomy (47). A patient with SHORT syndrome associated APDS2 was found to have pulmonary hypertension, but this was likely secondary to the presence of mitral stenosis, although significant respiratory infections were also present (18). A single patient with a PIK3R1 mutation was found to have tracheomegaly as well as megancephaly and a double aortic arch in the context of megalencephaly capillary malformation syndrome (8). Common airway diseases such as asthma have seemingly been observed only at low frequency [e.g., Ref. (26)], and it is difficult to draw conclusions from these occasional reports.

## Management of the Respiratory Manifestations of APDS

The majority of patients with APDS1 [87% (20) and 73% (8)] and APDS2 [89% (22)] are reported as receiving immunoglobulin replacement, often from an early age; this high proportion exceeds the numbers reported to have low IgG levels, suggesting that the drivers for commencing therapy include recurrent infections in the setting of specific antibody deficiency or subclass deficiencies. More patients in the APDS1 cohorts (62 or 63%) than the APDS2 cohort (17%) noted above received additional prophylactic antibiotics (most commonly co-trimoxazole or azithromycin); the reason for this difference is unclear. There are little available data on the efficacy of these interventions; Coulter et al. (20) stated that there was “reported benefit in most cases,” with none of the other case series specifically addressing this issue. Case reports have suggested that some patients exhibit marked (14, 23, 30, 36) or partial improvements (27), but others have flagged patients who had significant ongoing respiratory sepsis in the face of these treatments (15, 18). Of note, 42/68 patients currently listed on the APDS registry are currently receiving immunoglobulin replacement (see text footnote 1), with an overall reported decrease in respiratory infection and no withdrawals from therapy.

Rapamycin has been used to treat benign lymphoproliferative disease in APDS with some reported success (3), but respiratory-specific outcomes have not been published to date. A 12-week experimental medicine study (48) of the selective PI3Kδ inhibitor Leniolisib in six patients with APDS1 (three of whom had bronchiectasis) again did not report respiratory outcomes, but the observed improvements in B cell abnormalities characteristic of this disease (e.g., a reduction in circulating transitional B cells) suggest the potential for restoration of B cell function and hence a pulmonary protective role. Longer treatment regimens will be required to fully evaluate the benefits (and potential risks) of such interventions. Concerns have been raised that long-term PI3Kδ blockade increases genomic instability in B cells (49); however, these experiments were undertaken in mouse cells, and it is not clear that the same issues would complicate a therapeutic strategy aimed at normalizing, rather than abolishing PI3Kδ activity (48). Improvements in sinopulmonary infection have been reported following hematopoietic stem cell transplantation, with the majority of surviving patients no longer requiring immunoglobulin therapy (50); however, this procedure carries a significant mortality and will not alleviate established structural lung damage such as bronchiectasis. Early identification of patients with APDS (16) may allow transplantation before the development of such complications; however, the clinical heterogeneity makes prediction of future disease severity challenging.

## Conclusion and Outlook

Despite the varied clinical manifestations of APDS, respiratory infections are a near-universal feature and often predominate in the early phase of the disease. A number of mechanisms may lead to this enhanced respiratory susceptibility (Figure 1). Viral pathogens subvert host PI3K signaling, and this may contribute to recurrent upper respiratory infections and impaired airway epithelial defensive function. Compromised antibody production, perhaps combined with aberrant cytokine production and the viral-induced airway damage, contributes to increased susceptibility to bacterial pathogens and recurrent lower respiratory infections. Cycles of infection lead to permanent damage to the lower airways, with the development of bronchiectasis, and may further drive the benign lymphoproliferation that is a prominent feature of APDS. In addition to supportive treatment (with immunoglobulin replacement and prophylactic antibiotics), the use of PI3Kδ inhibitors has the potential for a highly personalized treatment strategy. The identification of biomarkers to predict specific complications and disease severity would be of value in selecting patients for potentially curative bone marrow transplantation.

## Author Contributions

Both authors contributed equally to writing this review.

## Conflict of Interest Statement

The authors declare that the research was conducted in the absence of any commercial or financial relationships that could be construed as a potential conflict of interest.

## Footnotes

^1^Maccari ME, Abolhassani H, Aghamohammadi A, Aiuti A, Aleinikova O, Bangs C, et al. Disease evolution and response to rapamycin in Activated PI3Kδ Syndrome: the ESID-APDS registry. (submitted to this Research Topic).
